# Debate: The impact of school closures and lockdown on mental health in young people

**DOI:** 10.1111/camh.12428

**Published:** 2020-10-13

**Authors:** Ellen Townsend

**Affiliations:** ^1^ School of Psychology University of Nottingham Nottingham UK

## Abstract

The COVID‐19 pandemic lockdown response has had a disproportionate and damaging effect on the lives, mental health and well‐being of young people globally. They have been neglected in policy‐making and their needs have been subjugated to those of adults which contravenes the UN Convention on the Rights of the Child. Here, I argue that the needs and rights of young people must come first to protect their health, mental health and futures. If we do not do this, we will let down a generation of children who will bear the brunt of the fallout of the economic burden of the global COVID‐19 crisis.

The rights and needs of children and adolescents have been ignored in the global pandemic crisis. This is a global disaster in the making, and frankly, we should be collectively ashamed of our neglect of young people during this time. Our children are the very people who will bear the brunt of the economic fall out of the pandemic lockdown and are also the ones being most damaged by it.

More and more the evidence shows that children are not significant in the spread of the virus and they do not (with very rare exceptions) suffer from the virus. What they have done is given up five months of their young lives, had hopes and dreams dashed, and been given shoddy examination results in return for their sacrifice. Enough is enough. Let kids be kids and let them get back to living normal lives, including going to school and taking part in after school activities such as drama, music, singing and sport. As my nine‐year‐old daughter put it to me recently, ‘We need to be able to learn and play with our friends – do all the normal things we do at school’. Normal means face‐to‐face playing and socialising, which is crucial for healthy development, for tots to teens.

Evidence increasingly shows that the lockdown has had a profound influence on the education, well‐being and mental health of many of our youngsters – and this impact will be long term, lasting many, many years. As world‐leading developmental psychologist and neuroscientist Professor Uta Frith put it recently, writing for reachwell.org (leading scientists providing evidence briefings on the impact of lockdown) ‘Education changes the way we perceive the world and behave in relation to others, and this affects our brain directly'. (Frith, 2020). The consequences for child development in the years to come could be vast, with impacts likely on self‐control, social competence and logical deduction amongst other cognitive abilities.

The impact of missing out on formal learning and the already embarrassing attainment gap in our education system is likely to worsen even with the measures proposed by the government to counter learning loss. Remember that so‐called ‘summer learning loss’ (experienced over the summer holidays) is an issue that used to be a major concern for teachers. (Indeed, all parents desperate for a cheaper summer family holiday outside of peak season have been strictly warned in the past of the dangers of even one day missed from school.) Most of our young people missed out on at least four months of formal education in 2020.

From the start of the lockdown, researchers working in mental health, self‐harm and suicide were worried about the effects of social isolation (Gunnell, 2020). Loneliness is as damaging as smoking and obesity in terms of long‐term health effects and is a significant risk factor for suicidal behaviour (McClelland et al., 2020). Research from the University of Oxford shows that young people have been feeling lonely in lockdown, and lonelier than their parents (ARC, 2020). University of Bath research indicates that loneliness is linked to mental health problems such as depression and anxiety up to nine years later (Loades, et al., 2020). An important new study from Cambridge indicates that ‘increases in stress across the entire population due to the coronavirus lockdown could cause far more young people to be at risk of suicide than can be detected through evidence of psychiatric disorders’. (Polek et al., 2020).

Even before lockdown, mental health problems and self‐harm were on the increase in young people. The risk of both self‐harm and suicide dramatically increases in the teenage years (the steepest increase in risk for any age group). During lockdown, we have asked young people to live in conditions that have exacerbated key risk factors for self‐harm and suicide ideation, including social isolation, loneliness, family problems and feeling trapped, defeated and hopeless.

We are already seeing increases in loneliness, behavioural and mental health problems in children and adolescents, and we know that economic downturns increase poor mental health and suicide rates (Reeves et al., 2012). Suicide is the leading cause of death in England in 5–19 year olds and it is worth remembering that more young people will die from suicide and road traffic accidents than COVID‐19 this year. Such bigger picture thinking has been curiously absent in policy‐making throughout this crisis, which has infuriated many academics who understand and work with risk. Take yourself out of the pathway of one harm and may put yourself directly in the path of another which could be much more dangerous. A classic example of this was when people switched to driving from flying around the United States after the 9/11 terrorist attacks, believing driving to be safer than flying (it wasn’t). The result was increased road traffic deaths.

Teachers worried about their health on returning to school can be reassured by data from the Office for National Statistics indicating that their profession is no more dangerous than others in terms of dying from COVID‐19. More evidence has emerged recently that schools do not pose a significant risk in transmission of the virus and the risk of children dying from COVID‐19 compared to other risks is very small (Bhopal et al., 2020).

So, the virus poses little risk to children, they don’t spread it to any great degree, and teaching is as safe as other professions in terms of risk from the virus. It is good news, then, that that children are back to school, but they must stay there. This is imperative to protect their development, mental health, well‐being and futures. Unfortunately, whole year groups are already being asked to quarantine for two weeks because one child has tested positive for COVID‐19. Their sacrifice has been enormous during this crisis and we should not ask them to give up any more. Lockdowns are a damaging luxury of which, Professor Sunetra Gupta (Professor of Theoretical Epidemiology at Oxford) said ‘I personally think that only thinking along the lines of eliminating coronavirus, without giving heed to the consequences on the disadvantaged young and globally, is a dereliction of our duties as global citizens’.

The UN Convention on the Rights of the Child (article 3) states: ‘In all actions concerning children, whether undertaken by public or private social welfare institutions, courts of law, administrative authorities or legislative bodies, the best interests of the child shall be a primary consideration’. The utterly shocking neglect of our young people in this crisis must now be put right. We need to put them first or be judged as letting down a generation of young people when we should have known better.

## Ethical information

No ethical approval was required for this article.
